# From Environmental to Possible Occupational Exposure to Risk Factors: What Role Do They Play in the Etiology of Endometriosis?

**DOI:** 10.3390/ijerph18020532

**Published:** 2021-01-11

**Authors:** Lidia Caporossi, Silvia Capanna, Paola Viganò, Alessandra Alteri, Bruno Papaleo

**Affiliations:** 1 INAIL, Department of Occupational and Environmental Medicine, Epidemiology and Hygiene, via Fontana Candida 1, 00078 Monte Porzio Catone, Italy; s.capanna@inail.it (S.C.); b.papaleo@inail.it (B.P.); 2 IRCCS San Raffele Scientific Institute, Reproductive Sciences Laboratory, Obstetrics and Gynaecology Unit, via Olgettina 60, 20132 Milan, Italy; vigano.paola@hsr.it; 3 IRCCS San Raffaele Scientific Institute, Obstetrics and Gynecology Unit, via Olgettina 60, 20132 Milan, Italy; alteri.alessandra@hsr.it

**Keywords:** endometriosis, endocrine disrupters, effects at low doses, workers, night shift

## Abstract

Endometriosis is a gynecological disorder characterized by the presence of endometrial stroma and glands outside the uterine cavity. A systematic review of the literature was conducted to clarify, starting from environmental exposure data, whether possible occupational risk factors may correlate with the onset of the disease. The guidelines for reporting systematic reviews of the “PRISMA” statement were followed and two databases, Scopus and PubMed, were used. Of the 422 studies selected with specific keywords, 32 publications were eligible, 28 of which referred to chemical agents and 4 related to night work. Conflicting data emerged among these studies. Although some compounds seemed to be more involved than others in the onset of endometriosis. Association with exposure to organochlorine compounds is the most supported by the epidemiological data, while other pesticide exposure did not show any clear correlation. Likewise, the hypothesis of a correlation with perfluoroalkyls exposure is not currently supported by data. The involvement of metals as risk factors has not been confirmed, while the role of night work, in the case of long service, seems to play an etiological role. In order to clarify the potential occupational risk of endometriosis development, well-designed studies are needed to evaluate the potential association between chemical compounds and disease etiology.

## 1. Introduction

Endometriosis is a common, often chronic, inflammatory condition characterized by the presence of endometrium outside the uterus, mainly on pelvic organs and tissues. Endometriosis is associated with severe pelvic pain as well as infertility [1]. It is estimated that approximately 10–15% of the female population is affected by this disease, and out of this percentage about 3% show conditions clinically relevant for women of reproductive age [2].

The exact etiology of endometriosis is unknown [3].

Several factors such as the genetic and immunological profile, the local inflammation, the hormonal activity and the metabolism of prostaglandins have been suggested to be involved in the onset and/or development of endometriosis [4]. From an epidemiological point of view, numerous hypotheses have been proposed and various investigations conducted to clarify the etiology of this disorder [5,6].

The possible role of environmental risk factors [7,8,9] or occupational risk factors [10] was investigated by some authors, highlighting contexts and exposures likely to be associated with the onset of the disease. There are two areas of potential risk that play a prominent role: exposure to chemical agents (particularly chemicals with xeno-estrogenic molecular activity [11]) and night work (linked to the interference on hormonal balance) [12].

To summarize the literature data available to date, a systematic review of published studies focused on occupational and nonoccupational population exposed to specific risk conditions has been herein conducted. 

## 2. Materials and Methods

### 2.1. Literature Search Methodology

This systematic review was conducted following the guidelines for reporting systematic reviews and meta-analyses—the “PRISMA” statement [13]. A literature search of relevant papers was conducted up to 27 February 2020 using the electronic bibliographic database Pubmed and Scopus. 

The search strategy included the keywords in the title or abstract; they were an association of “endometriosis” (first key word) and, as a second key word, “workplace”, “occupational exposure”, “endocrine disrupters”, “workers”, “chemical” and “night shift”.

### 2.2. Eligibility Criteria and Study Selection

We performed a search of papers published between January 1995 and February 2020. Only studies published in Italian, English and Spanish and reporting original data regarding the exposure to potential occupational-related risk factors, and epidemiological investigations related to endometriosis, were included. 

Strictly experimental studies, in vivo or in vitro, reviews and meta-analyses were excluded.

**Figure 1:**
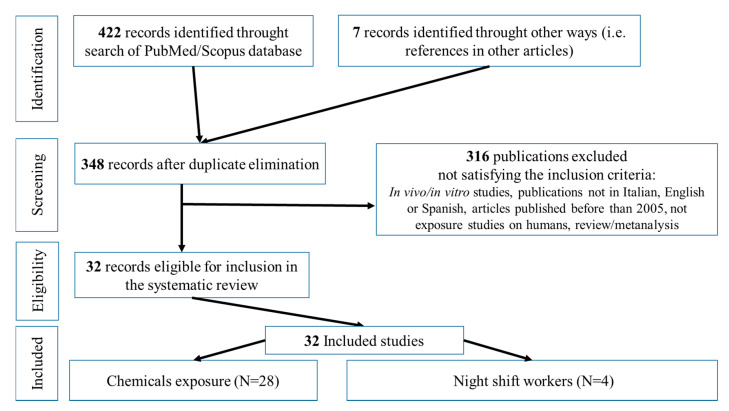
Selection process for articles.

The selection process of the articles used in this scientific review is schematically shown in Figure 1.

Eligibility assessment was performed independently in an unblended standardized manner by 2 reviewers. Disagreements between authors were resolved by consensus. One author, independently, extracted data from reports, using a predefined data field.

The epidemiological surveys taken into consideration are on general population, referring to chemical exposure, and retrospective studies (starting from an ill population of women, looking for possible health risk factors)—both cross sectional and case/control. No studies were published on the risks of endometriosis in the case of chemical exposure in the workplace, and this review aims to analyze the need to proceed with the execution of this type of investigation.

Only in the case of night shift were female workers enrolled, and the studies were in specific workplaces.

In search for epidemiological studies, the exposure to endocrine disruptors has been considered. 

These substances are able to interact at different levels with the endocrine system, therefore affecting reproductive health. In particular [14,15], the classes of substances for which a possible correlation was demonstrated with the incidence of endometriosis are plasticizers (specifically phthalates and alkylphenols); organohalogen compounds (organochlorine pesticides, perfluoroalkyls—PFAS); organophosphorus and pesticides, and some metals. 

There is also substantial literature [16,17,18,19,20,21] on the effects of environmental exposure to dioxins and polychlorinated biphenyls on female reproductive health, including endometriosis. However, since these substances are banned from industrial production, and consequently occupational exposure is impossible, we decided to exclude them from this review.

### 2.3. Bias Assessment

We assessed the possibility of bias across studies (publication bias) by evaluating a Funnel plot of the mean results’ differences for asymmetry, which can result from the nonpublication of small trials with negative results. We acknowledge that other factors, such as differences in study quality or heterogeneity, could produce asymmetry in Funnel plots. For each paper, we plotted the mean result by the inverse of its standard error.

The assessment of bias within studies was carried out considering the Cochrane risk of bias tool [22]. Among the six types of bias proposed by the tool, we used, for the present systematic review, only the last three (incomplete outcome data, selective reporting, other bias). This was because the other 3 were linked to a possible clinical trial (selection bias linked to random sequence generation and allocation concealment; performance bias linked to blinding of participants and personnel; detection bias linked to blinding of outcome assessment), not useful for the epidemiological studies collected for this review. 

**Figure 2:**
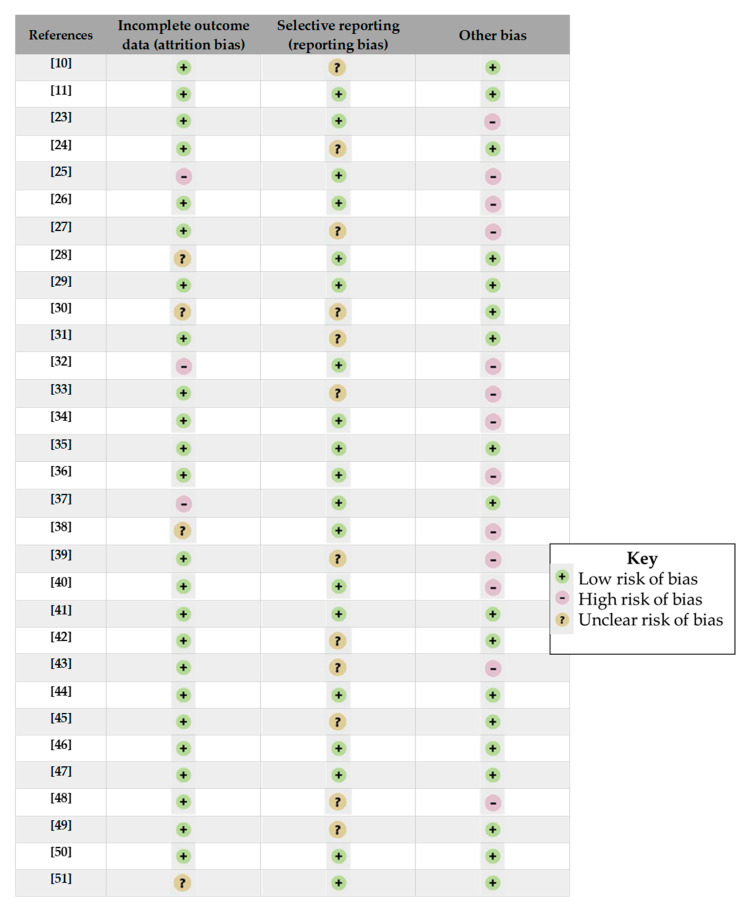
Bias assessment within articles [10,11,23,24,25,26,27,28,29,30,31,32,33,34,35,36,37,38,39,40,41,42,43,44,45,46,47,48,49,50,51].

In “other bias”, we considered the sample size, the number of controls, the source of data (biological analysis or self-reported information). The results of this evaluation are presented in Figure 2.

## 3. Results

### 3.1. Exposure to Plasticizers/Plasticizing Agents

Phthalates, dialkyl esters or alkyl aryl esters of orthophthalic acid (1,2-dicarboxylic acid) are plasticizing agents used in the production of numerous objects of daily usage. This is a group of structurally similar molecules, widely used in industry since 1930. In particular, di-(2-ethylhexyl) phthalate (DEHP) is a substance globally produced in quantities exceeding 2 million tons, and its dangerous characteristics, associated with reproductive toxicity, have been documented. Other important phthalates, in terms of industrial production, are diethyl phthalate (DEP), dibenzyl phthalate (DBzP), n-butyl phthalate (DnBP) and butylbenzyl phthalate (BBzP). Particularly for DEHP exposure, in vitro studies suggested its ability to activate aldo-keto reductase in the endometrium, possibly influencing the development of endometriosis [52].

The most representative alkylphenol is Bisphenol A (BPA) that acts also as an endocrine disruptor. This monomer is the building block of polycarbonate plastic and a component of epoxy resins used in numerous applications such as thermal receipt paper. It has xeno-estrogenic activities. Exposure to this chemical agent can occur during occupational activities or following migration from plastic containers into food. In the class of alkylphenols, octylphenol and nonylphenol are also represented. Bisphenol F and S have recently been used as substitutes for BPA, whose deleterious effects on health are starting to be known [23].

In the European context, some of these substances have been included in the list for REACH authorization, while others are subjected to restrictions due to the epidemiological evidence emerging in relation to their employment. Nevertheless, some substances such as DEP, are still used without restrictions, essentially in cosmetic products. Table 1 describes the characteristics of the considered studies investigating the exposure to phthalates and/or alkylphenols in relation to the incidence of endometriosis. 

Cross-sectional investigations do not support a higher risk hypothesis, identifying [31] a risk index equal to OR = 1.36 (95% CI 0.77–2.41) for MnBP and even a reduced risk (OR = 0.44, 95% CI 0.19–1.02) for one of the DEHP metabolites. Considering the case-control investigation on phthalates, which included the highest sample size [11], a possible correlation was identified between exposure to phthalates, particularly DEHP and MnOP, and the incidence and severity of the disease. In small samples, but with an adequate number of controls [28,29,30], with 97 cases and 169 controls, 85 cases and 135 controls and 92 cases and 195 controls, respectively, correlations emerged, more evident for BBzP, DnBP, DnOP and less for DEHP.

Investigations relating to BPA exposure, once again, conflict with respect to the results. The only cross-sectional study [24] (on 166 subjects) did not conclude with a hypothesis of correlation between exposure and disease. The numerically most significant case-control study [35] conducted on 143 cases/287 controls confirmed the lack of association between exposure to BPA and endometriosis. The other surveys presented such small samples that the results, which nevertheless conflicted with each other, were not very indicative. 

### 3.2. Exposure to Metals

Albeit inconsistently, some metals were shown to have harmful characteristics potentially interfering with the endocrine system, among which cadmium [53], lead and mercury [54] play a higher role. [55].

Cadmium compounds are commercially produced as fertilizers, stabilizers in the production of plastics and pigments and nickel-cadmium batteries. Cadmium is produced in the refining of zinc ore and some lead and copper ores [56]. Human exposure to cadmium can occur in living environments through the food chain [57,58] and smoking [59]. Possible occupational exposure to cadmium arises as an additional source of specific risk.

Lead compounds were widely used in various production processes during the twentieth century. Evidence of deleterious effects of this metal on health were recorded back in the early twentieth century, so restrictions on its use were gradually put in place. A recent study [60] was conducted to identify the main circumstances characterized by an occupational exposure to lead. The following contexts were investigated: welding; construction activities, especially in the case of renovating old buildings, plumbing works; radiator repair; metalworking and lead alloys; mining; lead–steel welding; indoor shooting range activities. More specifically, Koh et al. [61] reviewed the exposure data in a lead-based workplace from 1940 to 2010 and identified welding, handling and cutting of metals in general and the production of lead acid batteries as the activities with the highest risk, in addition to construction activities.

The use of mercury has been greatly reduced in production activities and highly regulated in the fields where it is still permitted. Indeed, its harmful effects, particularly of neurotoxic nature, have been evident since the end of the nineteenth century [62]. Historically, human exposure to this metal has occurred due to contamination of the food chain, especially methyl-mercury contamination of fish products, or in specific work contexts such as mining. More recently, the workplaces that are still potentially involved in this type of exposure are the following: orthodontics and dental activities, due to the use of mercury-based amalgams [63,64,65]; chlorine production plants, in which large quantities of mercury are used (100 tons to produce 50,000 tons of chlorine) [66]; the disposal and recycling of fluorescent lamps [67,68]; and gold mining, especially in developing countries [69].

Many of the potentially risky activities mentioned have been the prerogative of men for decades. Nevertheless, in recent years, we have witnessed a greater involvement of women in all work activities, and this requires particular attention from occupational doctors for the typically female risks. Table 2 shows the epidemiological investigations dealing with exposure to metals in relation to the onset of endometriosis.

### 3.3. Exposure to Organohalogen Compounds and Pesticides

Possible associations between exposure to organohalogen compounds (organochlorine pesticides [16] or perfluoroalkyls [7] in particular) or other classes of pesticides (organophosphorus, pyrethroids and phenoxy herbicides [15]) and endometriosis have been investigated. These studies confirmed the possibility that the agriculture activities may represent a risk setting in the event of exposure to a female population, due to the use of pesticide treatments based on organochlorine, organophosphorus, phenoxy herbicides or pyrethroids.

As far as perfluoroalkyls are concerned, apart from possible environmental contamination, they can be found in numerous commercially available products, such as lubricants, paints, cosmetics, some shampoos, fire-fighting foams, food containers, wrapping for microwave popcorn and some fabrics [7]. All the industrial contexts in which the substances, or materials containing the substances, are managed are considered to be potentially at risk of exposure to these chemical agents.

The studies concerning the exposure to pesticides and/or organohalogen compounds and the onset of endometriosis are shown in Table 3.

The cross-sectional investigations highlight a possible higher risk of endometriosis among exposed women. The only case-control study [44] with a numerically suitable sample (248 cases and 538 controls) confirms a greater risk of endometriosis, especially for those exposed to β-hexachlorocyclohexane. 

### 3.4. Night Workers

The significant and protracted alteration of the circadian rhythm over time was documented as affecting the daily levels of melatonin [70]. Melatonin seems to be inversely associated with estrogen hormones [71] and biochemical evidence supports emerging data, from population surveys, on the association between shift work and reproductive health.

In women, shift work, especially night work, impacts reproductive health, [72,73,74] in terms of increased numbers of menstrual disorders [75], miscarriages [76], low birth weight or preterm birth [77]. 

Data regarding the association between night work and the incidence of endometriosis are described in Table 4.

The outcome of the only cross-sectional study [49] does not show a higher incidence of disease in the event of exposure to night work. Only the subgroup of flight attendants, employed in long-distance nonstop flights, revealed a higher risk index (OR 2.2, 95% with CI 1.1–4.2), but important confounding factors have also been clearly referred to, such as exposure to cosmic rays, which can affect the final data.

The highest risk indices were proposed in particularly limited sample numbers. Marino et al. [10], for example, identified an OR in three categories of workers (9.80 for flight attendants, 5.77 for workers in the healthcare setting and 1.49 in workers at service stations, respectively). From an analysis of the sample, it was observed that 5 flight attendants were compared to only one control, yet the sample of healthcare workers consisted of 29 cases and 55 controls, while there were 4 service station workers and 2 controls. These numbers in no way allow us to consider the risk indices proposed as robust or extendable to larger samples.

The case-control study with a suitable number for an incidence of disease [50] (235 cases and 545 controls) is suggestive of a correlation between night work, namely, work activities that include night work at least half the working time, and the incidence of endometriosis. However, these data were not confirmed by the prospective study, conducted on 89.400 women with a follow-up of 16 years [51] whereby evidence of an association was upheld exclusively in the subgroup of nurses with work activity exceeding 5 years with concurrent infertility (OR 1.17, 95% CI 1.18–2.49); in this subgroup of female workers, the confounding factors could also be numerous, in addition to other possible occupational risk factors.

## 4. Discussion

The scientific studies collected do not show a particularly defined picture of the possibility of risk.

From a methodological viewpoint, criticisms that emerge in many of the studies taken into consideration are associated with the sample size in general and especially the number of controls in case-control investigations.

Considering a 10–15% prevalence of endometriosis in the female population of childbearing age [78], a minimum sample size allowing us to obtain significant data should consist of about 200 subjects.

The number of controls represents the second critical element. In a case-control study, the number of controls should be at least equivalent to the number of cases, although a higher number or even the doubling would be ideal, so that the results of the comparison would be more robust and, therefore, generalizable to other situations and contexts.

In consideration of the aforementioned, the most robust studies are those related to exposure to pesticides and organohalogen compounds. As to exposure to other chemical compounds or night work, very few studies met the indicated criteria.

When the various risk factors and their outcomes were considered in detail, conflicting findings were found among the investigations with respect to exposure to plasticizers; evidence of the correlation between exposure and disease does not emerge clearly [79,80]. Many studies were characterized by a small sample size and thus were difficult to generalize.

In addition, regarding DEHP analysis, many studies proceeded with the determination of a single metabolite. This entails a probable underestimation of the exposure and, therefore, the correctness of the assessment should be considered with caution. It should be further underlined that MEHP (the main metabolite chosen to track exposure to DEHP) alone does not represent the best choice due to the susceptibility to possible contamination [81].

The choice of some authors to proceed with a blood assay [33] of the analytes of interest is an important element of bias in plasticizers. Most of the laboratory consumables are made of plastic, containing different percentages of some phthalates and/or alkylphenols, and they can cause a contamination of the sample and, therefore, a possible overestimation of the findings [80]. On the other hand, the choice to proceed with the biological monitoring based on the determination of urinary metabolites allows us to overcome this problem, producing data that are certainly more truthful regarding the potential exposure of the subject.

Meta-analyses [14,82], as well as the systematic reviews [80,83] present in literature, fail to identify elements with a clear correlation. For some metabolites of DEHP, higher risk indices have been suggested, but various methodological limits have been claimed as involved.

The descriptive investigations of exposure to metals appear to be more homogeneous in the outcomes. Cross-sectional studies hypothesized an involvement of cadmium [37] and lead [42] in the onset of the disease. However, data were collected using self-reported questionnaires; hence, possible biases cannot be excluded. However, none of the case-control studies support this hypothesis, for any of the metals tested, especially in regard of cadmium, lead and mercury. Once again, the sample size is generally small and data must be interpreted very carefully.

Investigations related to pesticides and organohalogen compound exposure are, numerically, the most consistent. Exposure to organochlorines seems to be associated with the incidence of endometriosis. Cross-sectional studies [43,45] suggest their role in the etiology of endometriosis, particularly organochlorine fungicides and β-hexachlorocyclohexane and γ-hexachlorocyclohexane. Recently published reviews of scientific literature on the subject [14,16] confirm this hypothesis. The survey [47] on exposure to perfluoroalkyls suggests that these compounds may play a role in the onset of the disease, although the evidence of a single study, on 54 women with endometriosis, is not very indicative. Furthermore, regarding these compounds, some authors [84] suggested the role of oral contraceptives as confounding factors. However, what has been highlighted does not support the hypothesis of correlation between exposure to other types of pesticides, such as organophosphorus, pyrethroids and phenoxy herbicides [47], and endometriosis development, especially following the application of appropriate corrections for the confounding factors identified.

A final consideration about endocrine disruptors is needed: the dose/response curve for these chemicals was studies, and in numerous cases, a nonmonotonic trend was found [85]; this means that, even at low doses, biological effects could be relievable, sometimes comparable with higher doses. This topic must to be considered, together with the possibility of the multiple exposure to different chemicals at low doses.

Overall, the study of the published surveys raises doubts on the correlation between night work and the incidence of endometriosis. The results that emerged are not conclusive and demonstrated some elements of uncertainty.

Therefore, the association between night work and the incidence of endometriosis cannot be clearly identified, as data are conflicting, all the possible confounding factors were not properly evaluated and the data are not unambiguous.

It is important to take into consideration the confounding factors that may still affect the onset of the disease. Tobacco smoking, in particular, was investigated as a potential risk factor for endometriosis development [86,87]. In a case-control study on 90 women with laparoscopically diagnosed endometriosis and 90 controls [87], the comparison between smokers and those who have never smoked resulted in an OR risk index = 2.36 (95% CI 1.04–5.35). Even more important was, in the comparison between smokers and nonsmokers, the stratification of the samples between nulliparous women and those who had given birth to at least one child. In this case, a higher risk was found for nulliparous women (OR = 2.42 95% CI 0.95–6.17). In a meta-analysis on the topic, in [87], 38 studies were considered for a total of 13.129 women diagnosed with endometriosis, no significant correlation between smoking and the incidence of endometriosis was shown.

## 5. Conclusions

Exposure to endocrine-disrupting chemicals can considerably affect female reproductive disorders. Exposure can take place in living environments, but also in specific workplaces in which these substances are produced or used.

The social and economic cost attributable to the negative effects of the exposure to endocrine disruptors on human health was estimated. For endometriosis and fibroids in particular, a cost of EUR 1.5 billion per year in Europe emerged. We would like to emphasize that this estimate has been made considering only those chemicals for which there are sufficient epidemiological studies supporting a causal link between exposure and effects on human health [88]. In light of these data, it is extremely urgent and appropriate to conduct studies in order to understand the etiology of endometriosis and to reduce its incidence, where possible. Any identification of occupational risk factors would require the active involvement of an occupational doctor in the prevention and assessment of the risk.

From a review of the scientific literature produced in the last 15 years regarding the onset of endometriosis and specific occupational exposure to risk factors, discordant and inconclusive elements emerge. 

Epidemiological data mostly support the organochlorine exposure as a risk factor for endometriosis development. Although for some compounds, such as DEHP, several authors suggest a possible involvement in the disease etiology, the characteristics of the published studies do not allow us to confirm these data with robust results. Of course, numerically consistent epidemiological investigations, case-control and prospective studies are needed, in order to support or refute this hypothesis

The role of night work (particularly if it consists of rotating nightshift work and for at least 5 years) seems to play some role in the etiology of the disease. However, confounding factors were not always properly considered.

It is desirable to carry out numerically useful surveys for statistical significance, with detailed considerations with respect to possible confounding factors (smoking, the use of oral contraceptives or other occupational risk factors) and the best methods of dosing the analytes to avoid overestimates due to contamination from laboratory material (for blood tests) or underestimates for the analysis of single metabolites.

## Figures and Tables

**Table 1:** Description of the investigations conducted with respect to phthalates and/or bisphenols exposure and endometriosis.

Ref.	Substance	Type of Study	Sample	Biomarkers and Concentrations	Results	Considerations
[24]	BPA ^a^	cross-sectional	166 women with endometriosis	Urinary BPA (μg/g creat. ^b^) 0.80 (median)	No correlation was found between urinary BPA levels and disease severity.	Mean BPA values did not differ significantly between women with stage 0–I (0.74μg/g creat.) of endometriosis severity compared to stage II–IV (0.93 μg/g creat.).
[25]	BPA and BPB ^c^	case-control	58 women with endometriosis, 11 women controls	Serum BPA and BPB (μg/L) BPA 2.91 vs. 0.00 BPB 5.15 vs. 0.00 (arithmetic mean)	None of the control biological samples showed the presence of BPA or BPB, whereas there was presence in 63.8% of cases.	The sample size and the absence of an evaluation of statistical significance make the data indicative but not usable for the purpose of an evaluation of correlation with onset of the disease.
[26]	BPA and phthalates	case-control	30 women cases, with laparoscopic diagnosis of endometriosis and 22 women controls	Urinary BPA and phthalate metabolites. (μg/g creat.) BPA: 9.78 vs. 8.80 MMP ^d^ 62.8 vs. 105.0 MiBP ^e^ 185.0 vs. 183.0 MnBP ^f^ 83.3 vs. 76.3 MCHP ^g^ 12.6 vs. 6.87 MEHP ^h^ 30.4 vs. 30.2 MiNP ^i^ 73.2 vs. 18.9 MOP ^j^ 670.0 vs. <LOQ MBzP ^k^ 23.8 vs. <LOQ (arithmetic mean)	Absence of statistically significant differences between the two groups. For monoisobutyl phthalate, an OR ^l^ = 1.93, 95% CI ^m^ 0.51–7.33) was obtained but not significant (χ^2^).	The sample size can greatly affect the consistency of the study outcome.
[23]	BPA, BPF ^n^ and BPS ^o^	case-control	35 women with laparoscopic diagnosis of endometriosis, and 89 women controls	Urinary BPA, BPF, BPS (μg/g creat.) BPA 3.6 vs. 3.0 BPS 0.1 vs. 0,2 BPF 0.1 vs. 0.1 (geometric mean)	There is an increased risk between: BPA exposure and endometriosis- OR 1.5, 95% CI 1.0–2.3; exposure to the sum of three alkylphenols (BPA, BPF, BPS) and endometriosis -OR = 1.5, 95% CI 0.9–2.3.	Exposure to BPA is suggestive of a greater risk of endometriosis, but the study sample is numerically limited.
[27]	phthalates	case-control	55 women with endometriosis, 33 women controls	Urinary phthalate metabolites (μg/g creat.) MEHHP ^p^ 18.2 vs. 12.9 MEOHP ^q^ 13.4 vs. 10.3 MnBP 41.7 vs. 32.4 MBzP 5.8 vs. 7.3 MECPP ^r^ 23.8 vs. 19.0 (arithmetic mean)	The association between MEHHP and MEOHP levels (both metabolites of DEHP ^s^) and endometriosis was significant.	The data were suggestive of a possible association between exposure and endometriosis, but the sample size can considerably affect the robustness of the study, in addition to the reduced number of controls compared to cases.
[28]	phthalates	case-control	85 women with laparoscopic diagnosis of endometriosis and 135 women controls	Blood phthalates. (μg/mL) DnBP ^t^ 0.44 vs. 0.15 BBzP ^u^ 0.66 vs. 0.11 DnOP ^v^ 3.32 vs. 0.00 DEHP 2.44 vs. 0.45 (arithmetic mean)	Statistically significant differences emerged between the controls and the group of women with stage I and IV of endometriosis severity; in particular for DnBP, BBzP, DEHP and DnOP (*p* <0.05 ANOVA ^w^).	The results were indicative of a correlation between exposure to some phthalates and the onset of endometriosis.
[29]	phthalates	case-control	92 women with endometriosis and 195 women controls	Urinary phthalate metabolites (μg/g creat.) MEHP 2.2 vs. 3.4 MEHHP 14.8 vs. 18.8 MEOHP 8.1 vs. 10.8 MBzP 4.5 vs. 5.0 MEP ^x^ 61.9 vs. 43.9 MECPP 14.4 vs. 18.0 MiBP 1.3 vs. 1.5 MnBP 9.8 vs. 10.0 (median)	Inverse association between urinary levels of the DEHP metabolites and endometriosis was found, while a direct correlation for MBzP and MEP was identified, both without statistical significance.	Controversial data in terms of risk for incidence of endometriosis emerged, different by type of phthalate.
[30]	phthalates	case-control	97 women with endometriosis and 169 women controls	Blood phthalates (μg/L) MEHP 17.4 vs. 12.4 DEHP 179.7 vs. 92.5 (arithmetic mean)	There was a statistically significant difference between cases and controls in DEHP blood levels (179.7 ± 32.5 ng/mL vs. 92.5 ± 31.1 ng/mL) with a weak but significant association with MEHP (OR = 1.020, 95% CI 1.003–1.038 *p* = 0.020).	In the stratification of the sample with respect to the stages of severity of endometriosis, the data suggest a correlation between exposure and disease. The blood determination of phthalates carries a greater risk of contamination by laboratory plastic material than the urinary determination of metabolites.
[31]	phthalates	cross-sectional	1227 women with self-reported diseases—of which 87 with endometriosis	Urinary phthalate metabolites (μg/L) MEHP 2.5 vs. 3.4 MBP 28.9 vs. 25.5 MEP 207.0 vs. 219.9 MBzP 14.4 vs. 14.1 MEHHP 16.5 vs. 19.7 MEOHP 11.5 vs. 13.5 (geometric mean)	An association between endometriosis and exposure to MnBP, OR 1.36 (95% CI 0.77–2.41) and an inverse association with MEHP OR = 0.44 (95% CI 0.19–1.02) were identified.	Need for confirmation of data in prospective and case-control studies.
[32]	phthalates	case-control	57 women with laparoscopic diagnosis of endometriosis and 80 women controls (stage 0–I endometriosis severity)	Urinary phthalates metabolites (μg/L) MnBP 26.5 vs. 20.0 MMP 12.0 vs. 8.3 MEHP 8.9 vs. 5.4 MEHHP 17.6 vs. 9.1 MEOHP 8.0 vs. 3.7 (arithmetic mean)	The comparison between the urinary levels of MEHP, MnBP, MBzP, MEHHP did not reveal a significant correlation between exposure to phthalates and endometriosis.	The results do not support the hypothesis of a greater risk of endometriosis in the case of greater exposure to phthalates.
[33]	phthalates	case-control	49 infertile women with endometriosis; 38 infertile women controls but without endometriosis; 21 fertile women without endometriosis	Blood phthalates (μg/L) Stage IV ofendometriosis severity vs. controls: DnBP 1.05 vs. 0.11 BBzP 1.27 vs. 0.14 DEHP 4.39 vs. 0.48 DnOP 5.35 vs. 0.03 (arithmetic mean)	The cases showed significantly higher values of DnBP, BBzP, DnOP and serum DEHP compared to the two control groups. Furthermore, the correlation between phthalates and the severity of endometriosis seemed statistically significant.	The study supports the hypothesis of correlation between exposure to phthalates and the onset of endometriosis, although the sample size is particularly low. The blood determination of phthalates carries a greater risk of contamination by laboratory plastic material than the urinary determination of metabolites.
[34]	phthalates	case-control	28 women with laparoscopic diagnosis of endometriosis, 29 women controls	Urinary phthalate metabolites (μg/g creat.) MnBP 94.1 vs. 58.0 MMP 52.4 vs. 32.1 MEP 58.0 vs. 71.4 MBzP 12.2 vs. 8.9 MEHP 4.2 vs. 3.4 5-oxo-MEHP ^y^ 19.0 vs. 7.8 5-OH-MEHP ^z^ 16.7 vs. 9.9 (arithmetic mean)	The cases showed higher urinary levels of MnBP (94.1 vs. 58.0 μg/g creat.) than controls.	The same authors suggested producing further studies of greater statistical strength to confirm the data relating to the possible association between exposure to phthalates and endometriosis.
[35]	BPA	case-control	143 women with laparoscopic diagnosis of endometriosis. 287 women controls	Urinary BPA (μg/g creat.) 1.32 vs. 1.24 (median)	Total BPA urinary levels did not show differences between cases and controls and no correlation with the different stages of severity of the disease was found.	Limitations of the study: the sampling time of the biological material (single urine spot sample); the absence of disease in the controls was self-declared, which could make the control sample not entirely suitable.
[36]	BPA	case-control	68 women with endometriosis and 60 women controls	Urinary BPA (μg/L) 5.31 vs. 1.64 (arithmetic mean)	A significant difference between cases and controls in BPA urinary levels was found. Conflicting data emerged with respect to the identification of work activities potentially at greater risk of endometriosis and/or exposure to BPA.	The data relating to work activity were collected with self-administration of a questionnaire. This generated mixed data. The authors underlined the need to structure exposure studies directly in specific work environments.
[11]	phthalates	case-control (matched cohort design)	495 women of “operative cohort” (190 with endometriosis) and 131 women of the “population cohort” (14 with endometriosis)	Urinary BPA and phthalate metabolites (μg/L) (operative/population cohort) BPA 1.5 vs. 1.6/4.2 vs. 1.7 MMP 2.1 vs. 2.4/3.7 vs. 2.7 MEP 107.2 vs. 109.6/ 152.0 vs. 138.2 MCPP ^A^ 2.7 vs. 3.4/ 5.8 vs. 4.1 MnBP 12.1 vs. 11.0/ 19.1 vs. 11.2 MECCP 24.7 vs. 22.0/54.2 vs. 20.3 MCMHP ^B^ 29.3 vs. 29.2/ 53.5 vs. 22.5 MEHHP 16.3 vs. 14.4/ 32.4 vs. 11.9 MEOHP 11.0 vs. 10.1/ 23.0 vs. 8.3 MCHP 0.03 vs. 0.04/ 0.04 vs. 0.03 MBzP 7.0 vs. 7.8/ 9.9 vs. 6.5 MEHP 4.8vs. 4.1/ 8.3 vs. 3.1 MOP 0.06vs0.06/ 0.06 vs. 0.05 MiNP 0.16vs0.16/ 0.22 vs. 0.16 (geometric mean)	Urinary levels of the metabolites of 6 phthalates were associated with an increase in the diagnosis of endometriosis and in particular for MnOP (OR 1.38, 95% CI 1.10–1.72) and MEHP (OR 1.35, 95% CI 1.03–1.78).	The greatest significance was observed in sub-populations including laparoscopic and cytological diagnoses.

^a^ BPA—bisphenol A; ^b^ creat. —creatinine; ^c^ BPB—bisphenol B; ^d^ MMP—monomethylphthalate; ^e^ MiBP—mono isobutylphthalate; ^f^ MnBP—mono-n-butylphthalate; ^g^ MCHP—monocyclohexylphthalate; ^h^ MEHP—mono(2-ethylhexyl)phthalate; ^i^ MiNP—mono iso-nonylphthalate; ^j^ MOP—mono n-octylphthalate; ^k^ MBzP—monobenzylphthalate; ^l^ OR—Odds ratio; ^m^ CI—confidence interval; ^n^ BPF—bisphenol F; ^o^ BPS—bisphenol S; ^p^ MEHHP—mono(2-ethyl-5-hydroxyhexyl)phthalate; ^q^ MEOHP—mono(2-ethyl-5-oxohaxyl)phthalate; ^r^ MECPP—mono(2-ethyl-5-carboxypentyl) phthalate; ^s^ DEHP—di(2-ethylhexyl)phthalate; ^t^ DnBP—di-n-butylphthalate; ^u^ BBzP—butylbenzylphthalate; ^v^ DnOP—di-n-octylphthalate; ^w^ ANOVA—analysis of variance; ^x^ MEP—monoethylphthalate; ^y^ 5-oxo-MEHP—mono(2-ethyl-5-oxo-hexyl)phthalate; ^z^ 5-OH-MEHP—Mono-(2-ethyl-5-hydroxyhexyl) phthalate; ^A^ MCPP—Mono (3-carboxypropyl) phthalate; ^B^ MCMHP—mono(2-carboxymethylhexyl) phthalate.

**Table 2:** Description of the investigations conducted with respect to metals exposure and endometriosis.

Ref.	Metal	Type of Study	Sample	Result	Considerations
[37]	cadmium, lead and mercury	cross-sectional	1425 women with self-reported endometriosis	A correspondence between blood levels of cadmium and endometriosis was highlighted; 2nd tertile vs. 1st OR ^a^ = 1.94, 95% CI ^b^ 0.73–5.18; 3rd tertile vs. 1st OR = 3.39, 95% CI 1.37–8.40.	It is a cross-sectional study; the data should be confirmed with prospective and case-control studies.
[38]	cadmium	case-control	54 infertile women with endometriosis and 74 women controls	No statistically significant differences emerged between urinary cadmium levels in cases and controls.	The hypothesis of interaction between cadmium and endometriosis was not confirmed; the sample was small.
[39]	cadmium and lead	case-control	119 women with endometriosis and 25 women controls	The mean urinary and blood values of cadmium did not differ in the two groups. The values of lead in the cases were lower than in the controls.	The data did not support the hypothesis of exposure to metals as a risk factor; the number of controls was particularly small.
[40]	lead, cadmium, nickel	case-control	50 women with laparoscopic diagnosis of endometriosis and 50 women controls	Blood data for cadmium and lead showed no differences between the two groups. On average nickel values were significantly higher in cases (2.6 vs. 0.8 μg/L).	The association between exposure to nickel and the onset of endometriosis is controversial for the same authors, especially due to the small study sample.
[41]	lead cadmium, mercury and other metallic elements	case-control	473 women with surgical diagnosis of endometriosis, 131 women controls without endometriosis highlighted with magnetic resonance imaging	Blood cadmium was inversely associated with the diagnosis of endometriosis (OR = 0.55, 95% CI 0.31–0.98), while urinary chromium and copper showed a direct correlation (OR 1.97, 95% CI 1.21–3.19 and OR 2.66, 95% CI 1.26–5.64, respectively).	The data did not support the hypothesis of a correlation between exposure to lead, mercury or cadmium and the onset of endometriosis. The number of controls compared to the cases was far too low to give robustness to the statistical processing.
[42]	lead, cadmium, mercury and other metallic elements	cross-sectional	190 infertile women, with and without endometriosis	The only significant data was for lead: OR = 2.59, 95% CI 1.11–6.06.	Infertile women with endometriosis had higher blood lead values than the others. The results require confirmation with prospective studies.

^a^ OR—Odds ratio; ^b^ CI—confidence interval.

**Table 3:** Description of the investigations conducted with respect to organohalogen compounds and/or pesticide exposure and endometriosis.

Ref.	Substance	Type of Study	Sample	Results	Considerations
[43]	organo-chlorinated	case-control	29 women with endometriosis and 51 women controls	The highest tertile of aromatic fungicide exposure was associated with a higher risk of endometriosis (OR ^a^ = 5.3, 95% CI ^b^ 1.2–23.6).	Increased risk due to aromatic fungicides exposure emerged; the sample number was very small.
[44]	organo-chlorinated	case-control	248 women with endometriosis and 538 women controls	Increased risk due to β-chlorocyclohexane exposure (fourth vs. first tertile OR 1.3, 95% CI 0.8–2.4) emerged. The association was greater between serum levels of β-chlorocyclohexane and ovarian endometriosis (OR 2.5, 95% CI 1.1–5.3).	The data were suggestive of an increased risk of endometriosis due to pesticide exposure, particularly for β-chlorocyclohexane.
[45]	organo-chlorinated	cross-sectional matched cohort	473 women (190 with endometriosis), 127 in the general population (14 with endometriosis)	In the operative cohort, γ-hexachlorocyclohexane showed positive association with endometriosis (OR = 1.27, 95% CI 1.01–1.59) and β-hexachlorocyclohexane in the population cohort (OR 1.72, 95% CI: 1.09–2.72).	Positive association between organochlorine compound exposure and the onset of endometriosis emerged, particularly for β- and γ-hexachlorocyclohexane.
[46]	Perfluoro-alkyls	cross-sectional	753 women, (54 with self-reported endometriosis)	Serum levels of perfluoroalkyls are significantly higher in women with endometriosis.	The data were suggestive of a possible association between exposure and endometriosis.
[47]	organo-phosphorics, pyrethroids, phenoxy herbicides	cross-sectional matched cohort	471 women (188 with endometriosis) and 123 in the general population (14 with endometriosis)	High exposure levels to diazinon, chlorpyrifos and chlorpyrifos methyl seemed to be associated with a higher incidence of endometriosis.	The risk incidence data, adjusted due to possible confounding factors reduce/eliminate statistical significance.
[48]	organo-chlorinated	case-control	44 women with endometriosis and 49 women controls	A correlation was found, in particular, for: β-hexachlorocyclohexane (logOR = 0.46, 95% CI −0.09/1.00), hexachlorobenzene (log OR = 0.72, 95% CI 0.13/1.31); the trans-nonachlor (log OR = 0.79, 95% CI 0.17/1.41); dieldrin (log OR = 1.95% CI 0.41/1.59); oxychlordane (log OR = 1.17, 95% CI 0.38/1.95); the cis-heptachlor epoxide (log OR = 1.68, 95% CI 0.78/2.58).	The data were suggestive of an involvement of organochlorine compounds in the etiology of endometriosis. However, the sample size was small.

^a^ OR—Odds ratio; ^b^ CI—confidence interval.

**Table 4:** Description of investigations conducted with respect to night/shift work and endometriosis.

Reference	Type of Study	Sample	Results	Considerations
[49]	cross-sectional	1945 female flight attendants and 236 female teachers	There was no difference between the two cohorts, OR ^a^ 1.0 (95% CI ^b^ 0.5–2.2). Higher incidence of endometriosis in flight attendants with long haul flights than in the lowest quartiles OR 2.2 (95% CI 1.1–4.2) emerged.	In addition to shift work, the greatest exposure to cosmic rays for flight attendants was evaluated.
[50]	case-control	235 women with surgical diagnosis of endometriosis and 545 women controls	Night work was associated with an increase in the incidence of endometriosis OR 1.48 (95% CI 0.96–2.29), in the case of jobs involving more than half of their work time during the night hours OR 1.98 (95% CI 1.01–3.85). High risk of endometriosis emerged mostly for those who worked for 5 consecutive years for more than 50% of the time in night work shifts (OR = 5.32 95% CI 1.21–23.0).	The influence of night work, especially if prolonged, on the incidence of disease was suggested.
[51]	prospective (follow up 16 years)	89,400 women; 2062 women with laparoscopic diagnosis of endometriosis over 16 years of follow-up	There was no correlation between night work and the incidence of endometriosis. In the sub-sample of infertile women, a significance was highlighted for those who had been working at night for more than 5 years, OR 1.71 (95% CI 1.18–2.49).	Women with seniority (nurses) > 5 years, with night shifts, had a greater risk in the case of concomitant situation of infertility.
[10]	case-control	341 women with laparoscopic diagnosis of endometriosis and 742 women controls	Higher incidence of endometriosis reported in flight attendants (OR 9.80, 95% CI 1.08–89.02) serving at service stations (OR 5.77, 95% CI 1.03–32.43), health workers (OR 1.49, 95% CI 1.03– 2.15).	The sample size of the subgroups, divided by work activity, was very small.

^a^ OR—Odds ratio; ^b^ CI—confidence interval.
